# Systematic Review of the Exposure Assessment and Epidemiology of High-Frequency Voltage Transients

**DOI:** 10.3389/fpubh.2016.00052

**Published:** 2016-03-29

**Authors:** Frank de Vocht, Robert G. Olsen

**Affiliations:** ^1^School of Social and Community Medicine, University of Bristol, Bristol, UK; ^2^School of Electrical Engineering and Computer Science, Washington State University, Pullman, WA, USA

**Keywords:** dirty electricity, HFVT, epidemiology, exposure assessment, electromagnetic fields, radio frequency

## Abstract

Conclusions of epidemiological studies describing adverse health effects as a result of exposure to electromagnetic fields are not unanimous and often contradictory. It has been proposed that an explanation could be that high-frequency voltage transients [dirty electricity (DE)] which are superimposed on 50/60-Hz fields, but are generally not measured, are the real causal agent. DE has been linked to many different health and wellbeing effects, and on the basis of this, an industry selling measurement and filtering equipment is growing. We reviewed the available peer-reviewed evidence for DE as a causal agent for adverse human health effects. A literature search was performed in the Cochrane Library, PubMed, Web of Science, Google Scholar, and additional publications were obtained from reference lists and from the gray literature. This search resulted in 25 publications; 16 included primary epidemiological and/or exposure data. All studies were reviewed by both authors independently, and including a re-review of studies included in a review of data available up to July 31, 2009 by one of the authors. DE has been measured differently in different studies and comparison data are not available. There is no evidence for 50 Graham/Stetzer (GS) units as a safety threshold being anything more than arbitrary. The epidemiological evidence on human health effects of DE is primarily based on, often re-used, case descriptions. Quantitative evidence relies on self-reporting in non-blinded interventions, ecological associations, and one cross-sectional cohort study of cancer risk, which does not point to DE as the causal agent. The available evidence for DE as an exposure affecting human health at present does not stand up to scientific scrutiny.

## Introduction

Although humans have been exposed to electromagnetic radiation from natural sources throughout history, it has only been in the last 50–100 years that exposure, especially in the developed world, has been ubiquitous (1, 2). People are exposed to electric and magnetic fields that can be characterized by their orientation, amplitude and time variation, or frequency content. In all cases discussed here, the fields are classified as “non-ionizing” because the field is at a low enough frequency that it cannot cause ionization of a molecule (3); this is in contrast to ionizing radiation including alpha, beta, and gamma radiation (4). Concern about human exposure to non-ionizing electric and magnetic fields has been expressed since the mid 1920s (5). Interest in the subject increased in the 1940s with the development of radar and the increase in commensurate human exposure to electric and magnetic fields orders of magnitude higher than those of natural sources (6) and has continued to the present. While electric and magnetic field exposures with many different characteristics have been studied, the most extensively studied have been magnetic fields at the 50–60 Hz power transmission frequencies and electromagnetic fields at radio frequency (RF) or microwave frequency. As a result of these studies, a number of exposure standards have been written by panels of scientists aimed to include an appropriate range of expertise and to have a balance of perspectives, and who are charged to consider the entire range of literature on the subject (7–10), although alternative interpretations of the same data exist (11).

The introduction of electricity into society has drastically changed society as a whole and dramatically improved the lives of people in contemporary developed and developing societies. In fact, electrification was listed as the greatest achievement of the 20th century by the US National Academy of Engineering (12). However, there have also been concerns expressed that human exposure to electromagnetic fields may have adverse effects on human health (13). Of most concern is increased cancer risk and based on all available literature at the time, both extremely low-frequency (ELF) magnetic fields and RF electromagnetic fields have been classified as possibly carcinogenic to humans (Group 2B) by the International Agency for Research on Cancer (IARC), while static electric and magnetic fields and ELF electric fields are not classifiable as to their carcinogenicity to humans (Group 3) (14, 15). Studies oriented toward other health outcomes have been conducted over the years [for example, see Ref. (16)]. Some individuals may be more susceptible than others to the EMF exposure and may suffer from “Idiopathic Environment Illness with Attribution to Electromagnetic Fields” (“electromagnetic hypersensitivity”), although this condition has not been verified in double-blind, randomized controlled trials (17–19) and seems, at least in part, psychological in nature (20). Nonetheless, some evidence of possible biological responses to provocation has been published (21, 22).

For non-ionizing fields nonetheless, it remains unclear what the possible biological mechanisms (other than electrostimulation at frequencies below approximately 100 kHz and thermal effects that can occur at higher frequencies; both of which are well understood) leading to adverse health effects could be (8, 23, 24). This makes it difficult to establish whether a causal association between electromagnetic radiation and some adverse health effects does exist or whether observed associations are somehow the result of some unconsidered confounding factor.

Indeed, although health effects, such as those associated with exposure to power-line frequency electric and magnetic fields, are reported (25) conclusions about causality are difficult (26). A possible explanation is that some other factor or common source of error not yet identified may explain these findings.

Given the suggestion of “some other factor,” Graham proposed that (27), “high-frequency voltages present on the electric power wires in homes, offices, schools, and factories should be considered a potential pollutant.” To justify this hypothesis, he noted that “high-frequency voltages can cause currents in humans either by direct contact or by capacitive coupling.” The fact that the frequencies are “higher” is relevant because at ELF, the amplitudes of both capacitively coupled currents and magnetically induced voltages are proportional to frequency. Hence, higher frequency fields with smaller amplitude may produce the same voltages and/or currents as lower frequency fields with larger amplitudes. Graham then developed instrumentation to measure the high-frequency voltages between hot and neutral wires at outlets of building wiring that consisted of a “ubiquitous” filter to remove the 50–60 Hz voltages and related harmonics followed by a standard voltmeter set on the AC voltage scale to measure the root mean square amplitude of the remaining voltage (28). Graham noted that voltages measured in early (further undefined) experiments with this device were in the order of tens to hundreds of millivolts, while the voltage induced in a 1-m square loop by a 0.2-μT, 60-Hz magnetic field normal to the loop was <0.1 mV. This difference was his basic rationale for considering this exposure to be worth examining. It should be noted that neither of these two scenarios specifically addresses the problem of the relation between these measurements and actual exposure of a human. This issue will be explored further in this paper. Later work by Graham resulted in an evolving measurement device more fully described in (29). In that publication, a peak detector followed by a DC voltmeter was substituted for the AC voltmeter because, “the effects of transient electrical fields on humans are related more to their peak amplitude….” The final design of this meter is described in its patent application and patent (30). Quoting from the patent, “after attenuation of the AC power signal, the remaining signal is differentiated, rectified and smoothed to provide a DC signal ….” The output of the device is described in terms of millivolts, and a typical range of output voltage “when sufficient pollution is present” is given as 200–2000 mV.

Following Graham’s hypothesis, discussions of this subject began to appear in the peer-reviewed literature. In 2003 [the first known reference in the literature to the connection between “dirty electricity” (DE) and potential health effects], Stetzer discussed DE at the International Conference on Electromagnetic Fields and Human Health in Kazakhstan (31). In 2004, the first known reference to the use of Graham–Stetzer (GS) units to quantify DE (32) was made. In these and a number of subsequent publications, it has been alleged that the scientific community with their focus on electric and magnetic field exposures at single amplitudes and single frequencies with and without modulation had been looking at the wrong etiological agent, and that this merely served as a weak proxy for the true exposure (DE). The proponents of this hypothesis claim DE is the cause of a host of outcomes ranging from cancer, diabetes (or aggravate diabetes by affecting insulin levels), and multiple sclerosis to chronic stress and suicide. In fact, it has been alleged that DE may be responsible for “most of the diseases of civilization” (33).

In addition to several peer-reviewed publications that will be discussed in this review, DE is also the topic of several books (see Supplementary Material).

The purpose of this paper is, therefore, to critically evaluate the available, peer-reviewed evidence for the connection between exposure to DE and any or all of these outcomes.

## Materials and Methods

An extensive literature search was performed in the Cochrane Library, PubMed, Web of Science, and Google Scholar (up to March 9, 2015) using “dirty electricity” and “high frequency voltage transients” as broad search terms to identify peer-reviewed publications that investigated this exposure metric in relation to human exposures and health outcomes, rather than the more commonly accepted electromagnetic field metrics. Reference lists of identified papers were scanned for additional peer-reviewed publications. Additionally, because the number of authors publishing on “dirty electricity” is relatively modest, additional searches were conducted in the search engines above on their names along with manual searches of the references for peer-reviewed papers on DE, while their personal or commercial websites were also scanned for additional references that were manually searched. All identified studies were scrutinized by both authors and references that mentioned DE but did not study DE specifically, and instead studied, for example, electrification in general (2), and studies only referring to DE in relation to engineering and power consumption were removed.

## Results

### Literature Search

A total of 26 publications were identified (Table 1). One paper was retracted by the journal because of absence of ethical approval (34). Of the 25 remaining papers, one review, by one of the authors of this paper, reviewed primary data published up to July 31, 2009, which included seven published, peer-reviewed journals papers (35). The literature base for the present review includes an additional 18 publications, published either as conference abstracts prior to the initial review cut-off or published after the July 31, 2009 cut-off date, and now includes the total evidence base of 12 peer-reviewed journal and conference abstract publications of which three are reviews and three are exposure studies only, seven conference abstracts, one editorial mentioning DE, and six letters to various editors.

**Table 1:** **Overview of peer-reviewed publications and conference proceedings on “dirty electricity”**.

Reference	Year of publication	First author^a^	Title	Publication type	Study design^b^	Health effects^c^	Exposure assessment^d^	Study size	Risk of bias^e^	Rationale risk of bias evaluation
**Exposure measurement studies**										
(36)	2009	Trushina	The monitoring of dirty electricity in a secondary school in Kazan, Republic of Tatarstan, Russia	Journal article	Exposure measurement study	No data	GS units (exposed 90–>2,000; control 4–70)	11 school areas in 1 school	NA	NA
(37)	2010	Gajda	Estimation of ambient electric fields generated by dirty electricity from compact fluorescent lamps	Conference proceedings	Exposure measurement study	No data	No new data	1 location (laboratory controlled)	NA	NA
(38)	2014	Richman	A pilot neighborhood study toward establishing a benchmark for reducing electromagnetic field levels within single family residential dwellings	Journal article	Exposure study	Exposure only	No new data	29 houses	NA	NA
**Hypothesis generation/confirmation**										
(39)	2003	Maret	Electrical pollution in the standard electrical wires and their influence on people	Conference abstract	Hypothesis	fibromyalgia, attention deficit disorder, chronic fatigue syndrome, Gulf War syndrome, diabetes mellitus, asthma, hypertension, immune dysfunction	No data	NA	Unclear	Hypothesis generating review Not enough information provided for assessment risk of bias
(40)	2011	Milham	Dirty electricity, cellular telephone base stations, and neoplasia	Letter to the editor	Hypothesis	Cancer	No new data	NA	NA	Description of hypothesis only
(41)	2011	Dode	RE: dirty electricity, cellular telephone base stations, and neoplasia	Reply to letter to the editor	Hypothesis	Cancer	No new data	NA	NA	Description of hypothesis only
(42)	2011	Milham	Attention deficit hyperactivity disorder and dirty electricity	Letter to the editor	Hypothesis	Attention deficit hyperactivity disorder	GS units (exposed > 2,000; control < 50)		NA	Description of hypothesis only
**Population studies**										
(43)	2004	Havas (*)	Teacher and student response to the removal of dirty electricity by the Graham/Stetzer filter at willow wood school in Toronto, ON, Canada	Workshop proceedings	School-based cross-over trial	Seasonal affective disorder/EHS/wellbeing, general health, coughing, asthma, medication use, flu, student classroom behavior	No new data	18 teachers [same as Ref. (32)]	High	Most likely not peer-reviewed Non-controlled Non-randomized Non-blinded Self-reported health assessment No statistical analyzes
(44)	2008	Havas (*)	Power quality affects teacher wellbeing and student behavior in three Minnesota schools	Journal article	School-based cross-over trial	Health and wellbeing, student classroom behavior, asthma and other respiratory problems, psoriasis	GS units (exposed 90–>2,000; control 16–150)	31 class rooms, 44 teachers	High	Single-blind Non-randomized Not controlled in same period Self-reported outcomes No statistical analyses
(45)	2008	Milham (*)	A new electromagnetic exposure metric: high-frequency voltage transients associated with increased cancer incidence in teachers in a California school	Journal article	Cross-sectional cohort	Cancers: total, malignant melanoma, thyroid, uterus, female breast	GS units (exposed > 2,000; control < 2,000) or GS unit years (exposed > 10,000 and 5,000–10,000; control < 5,000)	51 rooms; 137 teachers (16 cases with 18 cancers)	High	Evidence of biased comparison with external population No adjustment for important confounders Exposure only measured once and assumed to be stable
(46)	2014	Milham	Evidence that dirty electricity is causing the worldwide epidemics of obesity and diabetes	Journal article	Ecological	Body mass index (BMI), fasting plasma glucose, prevalence of diabetes	No new data	National comparisons	High	Ecological study Obvious and known risk factors ignored
**Case studies**										
(33)	2013	Milham	Dirty electricity, chronic stress, neurotransmitters, and disease	Journal article	Case study	Urinary dopamine, Urinary phenylethylamine	GS Units (exposed 11,190; control 39)	7 cases	High	Non-randomized Non-controlled Non-blinded Response shows classic intervention effect Only spot samples taken No statistical analyses
(34)	2014	Havas	Replication of heart rate variability provocation study with 2.4-GHz cordless phone confirms original findings	Retracted journal article			NA	NA		
**Mixed individual case and school populations**										
(47)	2004	Havas	Graham/Stetzer filters improve power quality in homes and schools; reduce blood sugar levels in diabetics, multiple sclerosis symptoms, and headaches	Conference proceedings	Case study	diabetes, multiple sclerosis, energy levels, reduced asthma and allergy medication use	GS units (exposed 170–800; control 13–33) or millivolts (exposed > 10; control < 10)	6 cases, 1 case family, 2 schools (22 staff and unreported); 1 case and 1 school previously reported (33)	High	Most likely not peer-reviewed Case studies Not blinded Not controlled
(48)	2004	Morgan, LL	High-frequency transients on electrical wiring: a missing link to increasing diabetes and asthma?	Conference proceedings	Case study	Blood glucose, asthma, chronic fatigue syndrome, fibromyalgia, foggy cognition, numbness on whole side of body, loss of sense of smell	Millivolts (exposed 10; control 4)	4 cases, 1 school with (37 children with asthma)	Unclear	Peer-review unknown Not enough information provided for assessment risk of bias
(32)	2004	Havas	Dirty electricity and electrical hypersensitivity: five case studies	Workshop proceedings	Case study	Electrohypersensitivity (EHS), Multiple sclerosis, diabetes mellitus (measured as insulin and plasma glucose), asthma, attention deficit hyperactivity disorder (AD(H)D), wellbeing/EHS, student classroom behavior	GS units (exposed 170–2,000; control 15–70) or millivolts (exposed 13–101; control 8–24)	4 cases, 1 school	High	Most likely not peer-reviewed Self-reported health effects Non-blinding of participants Non-blinding of researchers
(49)	2006	Havas (*)	Electromagnetic hypersensitivity: biological effects of dirty electricity with emphasis on diabetes and multiple sclerosis	Journal article	Case study	Blood sugar, multiple sclerosis	GS units (exposed 135–410; control 32–38)	4 cases [2 previously reported (33, 40)] 2 schools [previously reported (33, 40)]	No new data	No new data
**Reviews**										
(50)	2009	Johansson	Disturbance of the immune system by electromagnetic fields – a potentially underlying cause for cellular damage and tissue repair reduction which could lead to disease and impairment	Journal article	Review	Effects on immune system	No new data	NA	NA	No specific associations with DE described
(35)	2010	De Vocht	“Dirty electricity”: what, where, and should we care?	Journal article	Review	No new data	No new data	NA	NA	NA
(51)	2011	Havas	Wind turbines make waves: why some residents near wind turbines become ill	Journal article	Review/hypothesis	Ill health, wellbeing	No new DE data	NA	Unclear	No detail presented to evaluate risk of bias
(52)	2012	Rajendran	Beyond type 1 and type 2 diabetes mellitus – any type 3 and type 4 diabetes mellitus?	Conference abstract	Review/hypothesis	Diabetes mellitus, plasma glucose levels, genotoxicity	No new data	NA	NA	Review
(53)	2013	Pall	Electromagnetic fields act *via* activation of voltage-gated calcium channels to produce beneficial or adverse effects	Journal article	Review	No specific associations with DE described	No new data	NA	NA	Hypothesis description only
**Editorials and letters**										
(54)	2009	Morgan, JW (*)	RE: a new electromagnetic exposure metric: high-frequency voltage transients associated with increased cancer incidence in teachers in a California school, May 28, 2008; 51:579–586	Letter to the editor	Letter to the editor	NA	No new data	NA	NA	Addresses problems with (45)
(55)	2012	Stanwell-Smith	Darker nights and dirty electricity	Editorial	NA	NA	No new data	NA	NA	NA
(56)	2014	De Vocht	Refutation of dirty electricity hypothesis in obesity: epistemological arguments and trans-disciplinary study using an instrumental variable	Letter to the editor		NA	No new data	NA	NA	NA
(57)	2014	Milham	Response to “Refutation of dirty electricity hypothesis in obesity: epistemological arguments and transdisciplinary study using an instrumental variable” by Frank de Vocht and Igor Burstyn	Letter to the editor	Response to Ref. (56)	NA	No new data	NA	NA	NA

^a^(*) indicating publication was included in 2010 review in Ref. (35).

*^b^Duplicate reporting of case studies has never been reported by authors, and identification has been done here based on similarities in case descriptions*.

*^c^Wellbeing/electrohypersensitivity/seasonal affective disorder symptoms: sleep quality, mood, cold extremities, head pressure, joint stiffness, pain in joints or muscles, headache, anxiety, fatigue, painful teeth and gums, ringing in ear, irritability, dizziness, swollen hands/feet, sense of accomplishment and satisfaction, energy, frustration, and focus*.

*^d^Data from studies published previously are not repeated here. No attempt has been made to characterize the different measurement systems that each use “millivolts” as output*.

*^e^Risk of bias assessed as “low,” “unclear,” or “high” comparable to the Cochrane Collaboration Risk of Bias Tool for RCTs (http://handbook.cochrane.org/front_page.htm), with rationale in adjacent column*.

### Exposure Assessment

#### Dirty Electricity Defined

Dirty electricity is a form of electrical exposure, but is different than the other, traditionally used exposures for which the subject is immersed in or exposed to an electric and/or magnetic field or subjected to an injected contact current. First, DE is not defined as either an electric or a magnetic field or contact current, but rather most commonly as a voltage (the negative integral of the electric field) between the hot and neutral wires at an outlet of building wiring circuits. Second, it is most commonly defined as the output of a meter that incorporates the design in Graham’s patent (30).

More specifically, it is usually quantified as the output in GS units of a Stetzerizer^®^ Microsurge meter when plugged into an electric outlet. This meter has been commercialized by Stetzer Electric, Inc.^1^ According to this same website, the design of the Stetzerizer^®^ Microsurge meter is based on the Graham patent (30), and a GS unit is a measure of the voltage’s derivative with respect to time, dv/dt and is defined as 24 V/s.^2^ It is interesting to note that a 230-V rms 50-Hz voltage, or “normal electricity” of domestic appliances (in Europe) has a maximum value for dv/dt of 102,000 V/s or approximately 4,250 GS units. DE is a rapidly time-varying transient voltage and is also, and more neutrally, referred to as “high-frequency voltage transients (HFVTs).” DE has also been measured using an oscilloscope (33) or a multimeter set to peak to peak voltage with filtering to remove 60 Hz and its harmonics (49, 58). Little if any information is given in the literature that can be used to relate the readings of either to those of the Stetzerizer^®^ Microsurge meter. The Stetzer Electric website listed above does provide data that relate the output of the meter in GS units to single frequency input signals of varying amplitude. This does not, however, solve the problem of relating the responses to more complex inputs since the meter’s response to input voltages is non-linear.

The DE theory is at variance with the exposure metrics generally used in studies to investigate whether non-ionizing electromagnetic fields may be associated with increased health risks to humans, and as a result also with exposure metrics used in guideline documentation such as those defined by ICNIRP and IEEE. Nevertheless, it has been linked to a variety of human health and wellbeing effects, and both meters to measure DE and filters designed to block or reduce DE exposure have been offered for sale. The aforementioned Stetzerizer^®^ filters are designed to reduce HFVTs between the hot and neutral wires at the outlet by using a capacitor to insert a low impedance path between the wires over the relevant frequency range and are manufactured by Stetzer Electric, Inc.

#### Rationale for Determining Safe Levels of Dirty Electricity

Important for epidemiological exposure assessment is that, as described, the most common method found in the DE literature for assessing exposure to DE is to use a Stetzerizer^®^ Microsurge meter to measure DE at several power outlets in a room. Individuals present in rooms with DE levels >50 GS units are considered to be exposed to DE. Unexposed “control” individuals fall into one of two categories: (1) truly unexposed controls who have never been exposed to DE > 50 GS units and (2) previously exposed individuals who were considered controls after their environment was “cleaned” to below 50 GS units by introduction of Stetzerizer^®^ filters. Comparisons are then made between “exposed” and “unexposed” individuals.

It is important to investigate the basis for the claim that 50 GS units is the threshold for safe exposure to DE or HFVTs. At least some proponents of this threshold base their opinion on Russian standards [Dr. Milham, personal communication; (49)]. Nevertheless, despite extensive searches by the authors and the additional engagement of a reference librarian, a Russian standard that specifically addressed DE or HFVTs was not identified. Rather, Russian standards that cover the appropriate frequency range for occupational exposure were found in (59). From 10 to 30 kHz, the maximum permissible electric field exposure is 500 V/m for the full working day, while for 30–100 kHz, the maximum permissible electric exposure is 20,000 (V/m)^2^-h for the working day. For an 8-h day for 30–100 kHz, this would be equivalent to a continuous exposure to 50 V/m. A Russian standard that applied to the general public was not identified.

An examination of the DE literature does reveal some case studies where the 50 GS value is used as the threshold to classify exposure or non-exposure. One is related to changes in behavior and was reported in Ref. (60). Another case relates to measurable improvement in physical abilities (32). A third case study relates to measurable changes in body chemistry and can be found in (32). Other similar cases can be found in Ref. (32, 47).

Another possible source for the 50 GS threshold may be the claim (45) to have identified a dose–response between exposure to DE and cancer risk in a Californian middle school. However, even if this were a valid inference (in this paper, the evidence was re-examined), it does not appear that this result could be used as support for the identification of 50 GS units as a threshold between safe and unsafe exposures for (at least) the following reason: the dose–response described in Table V (45) correlates GS unit years (i.e., exposure over a prolonged period of time) and cancer prevalence rather than simply with GS units. Further, all (or nearly all) teachers worked at the school for 20 years or less. The lowest exposure category in the dose response relationship was 5,000 GS years or less. Hence, a teacher who worked at the school for 20 years would have to have been exposed to more than 250 GS units on average to be in a higher risk category. Teachers at the school <20 years would have required an even higher exposure. As such, these data do not seem to support the safe/unsafe exposure threshold of 50 GS units.

As a final possible basis for this threshold value, Havas (49) stated that “*Values (of high-frequency voltage transients) should be less than 50 and, for optimum effectiveness, less than 30 G/S units*” and gives as a reference a document, “Permissible levels of high-frequency electromagnetic pollutions’ voltage in a wire of industrial frequency alternating current” a sanitary-epidemiologic norm Confirmed by the Order of the Head State Sanitary Physician of the Republic Kazakhstan, November 28, 2003. Despite this document also being referenced by several websites and books, attempts to verify the existence of this document using an English language search, using the expertise of reference librarians and an inter-library loan request only resulted in a translated, draft document referring to a 50-mV permissible level for the frequency range 1–400 kHz using equipment not equivalent to that specified by Graham (30). This draft document included an amendment authored by David Stetzer, president of Stetzer Electric, a company that sells DE meters and filters, that 50 mV should be 50 GS units (61). If this document is indeed the original source of the safety threshold of 50 GS units, then this raises a number of problems. First, and most important, no rationale is given for selection of the permissible level. Second, this level was selected in November 2003, prior to essentially all of the research on DE reported in the peer-reviewed literature. Third, the reported frequency range is 1–400 kHz rather than the 10–100 kHz range for the Stetzerizer^®^ Microsurge meter. Fourth, although it is assumed that a 1–400 kHz filter is used for the measurement, no mention is made of differentiating, peak detecting or averaging the remaining signal. Only a millivoltmeter is mentioned. Hence it is not at all clear that the measurement mimics that of a Stetzerizer^®^ Microsurge meter. Most importantly, despite Stetzer’s note about a translation inaccuracy that 50 mV should be stated as 50 GS units, the measurement system described in the document is clearly designed to report, and is consistent with, a value in millivolts. It is unlikely that there was a simple translation error, and in fact the unamended document provides no basis for a 50 GS unity safety threshold level.

Finally, it is interesting to note that no laboratory studies, which demonstrate an effect or proposed biophysical mechanism to explain any of the health effects reported in literature (or more specifically the threshold for safety) and which are discussed in detail below, have to date been conducted. The current DE hypothesis falls short of the testing of hypothesized biophysical mechanisms which, although not strictly necessary, would, if available, considerably strengthen the proponents’ case.

#### The DE vs. Human Exposure Conundrum

As introduced above, the metric most often used to measure DE is the voltage between the hot and neutral wires at outlets in building wiring systems that has been processed by a Stetzerizer^®^ Microsurge meter. This measure does not account for the fact that there may be other relevant voltages (e.g., between hot and safety wires) and currents on these wires that may or may not be balanced (i.e., equal and opposite currents or charges). If it is assumed that the electrical charges on these two wires are balanced (i.e., charge on one equal to the negative of charge on the other), the measured voltage can be related to an electric field in the vicinity of the wires using electrostatic theory. One consequence of this analysis is that since the wires are closely spaced, the electric field decays rapidly away from the wires due to cancelation from the balanced charges (37), and the result is an electric field significantly less than any existing exposure standard. For example, at a distance of 20 cm from a typical household wiring cable of a 13-W compact fluorescent lamp, the largest DE-generated electric field (at 54 kHz; the peak of the lamp’s spectrum) was approximately 0.034 V/m. This compares to the ICNIRP standard exposure limit of 170 V/m. In part because of this result and in part because other relevant voltages or the degree to which they are balanced have not been characterized, it is not at all clear exactly how the measured voltage translates into a significant electric field to which any individual might be exposed. Further, any magnetic fields from the associated currents (if any) have been neglected because the current is not measured. In addition, the pertinent question is not whether the fields can be detected as shown in (28). Many very small fields can be detected by, for example, radio receivers. In fact, it is well known that devices, such as compact fluorescent light bulbs, cause detectable electromagnetic interference (62). Rather, it is whether the associated fields couple strongly enough to a human body to cause some replicable biological response; ideally in a dose–response manner (63). For example, ICNIRP describes that at 10 kHz, the basic restriction on the electric field in the body is 2.7 V/m (8).

Graham originally suggested that DE could be coupled to the body by direct contact or capacitive coupling (27). The former idea has been developed further by Havas and Colling (51). They recognized that because the electrical system incorporates connections to the earth (for purposes of safety), unbalanced currents (neglected in the voltage measurement of DE) can flow in the earth. Since the earth is a resistive material, this can result in small voltages between different parts of the earth. A human who touched two points at different potentials would have a current in the body proportional to the DE voltage and inversely proportional to the impedance of the system plus human. This current would be distributed through the body according to the location of the touch points and the shape and distribution of body resistance. However, although this argument makes theoretical sense, there is no hint in the literature about whether DE measured with a Stetzerizer^®^ Microsurge meter is in fact directly related to this kind of exposure. Capacitive coupling of DE voltages to a circuit that includes the human body has also been described.^3^ In this case, the current in the body would be proportional to the DE voltage and to the very small capacitance of the path through the air to the body and would be distributed through the body. Again, this argument makes sense. However, little quantitative information about this circuit is given in the DE literature aside from an estimate of body capacitance that is at least an order of magnitude too high. In either case, the real issue is not whether these voltages can, in principle, be coupled to currents in the body, but whether the resulting current inside the body is strong enough to cause a replicable biological response, such as the basic restriction, is described by ICNIRP (8).

Perhaps it is true that this one DE measurement is a surrogate for relevant fields to which humans may be exposed, but little effort has been put into quantifying this issue or into the question of determining personal exposure (or even tissue dose) itself, instead of “exposure” on the mains. To do this, it would be reasonable to follow a procedure similar to that described by Nadakuduti et al. (64). In this paper, instrumentation for measuring the incident fields as well as the induced fields in humans in the 10 kHz–1 MHz range from compact fluorescent lamps was developed and used to conduct measurements. Given that this frequency range overlaps with those used in DE studies this measurement device, or ideally one measuring the frequency range of DE specifically, would also be relevant to advance DE exposure research.

In summary, the exposure conundrum described here is that very little if anything is known about the relationship between the metric (i.e., some manipulated version of the voltage at an electrical outlet between hot and neutral wires) used to quantify DE and, the actual fields to which humans are exposed and the resulting personal exposure or dose from induced fields in a human or the equivalent circuit through which DE voltages might couple to the body to produce a personal exposure or dose.

### Epidemiology

#### Review

The previously published review by one of the authors (35) included five peer-reviewed journal articles available at the time (up to July 31, 2009). It concluded that none of the published peer-reviewed publications were of sufficient scientific quality to be useful to determine whether DE may be the causal exposure for any of the health effects described. The majority of available data at the time consisted of a series of case descriptions that were often re-used in other publications, and all of which were based on self-reported health effects and non-blinded exposure conditions making them extremely susceptible to placebo and nocebo effects (65). This review includes 26 publications, 5 of which were included in the previous review of the epidemiological evidence (35) and 3 of which were exposure assessment studies only. An overview of all available data for this review is presented in Table 1. Three additional conference abstracts describing human studies were identified that were published prior to the previous epidemiological review’s cut-off data but not included in it because they were not peer-reviewed in Ref. (32, 47, 48).

Although the authors have not reported when case studies had been reported in previous publications, it was possible to identify with a moderate to high degree of certainty that now in total 15 individual cases “exist”; 4 healthy subjects, 1 self-diagnosed as electrohypersensitive, 6 suffering from multiple sclerosis, and 4 with diabetes. All described case studies rely on self-reported health effects, non-blinding of exposure, and/or variable times of day when measurements were taken. Importantly, case reports are considered the lowest level in the hierarchy of evidence in evidence-based medicine and public health (66). They can have some use in hypothesis generation, but because they have no comparison group, there is high potential for bias, it is doubtful if the results are generalizable and they should not be used to infer causality (67). This is unfortunately what has been done in most of the DE literature (not necessarily by the authors themselves, but subsequently in letters, hypothesis descriptions or reviews). Similar methodological problems were present in all of the School-based interventions (Table 1). If it is assumed however, that these studies were indeed only used to generate a new hypothesis, then subsequently no attempts have been made to investigate if any of the hypotheses generated from the case descriptions stands up to scientific scrutiny using a robust epidemiological study design; i.e., the double-blind, randomized, and controlled trial.

One trial has been conducted since the previous review, but this can be best described as a non-randomized, non-controlled, and non-blinded intervention (33). Scientific issues as well as issues with missing conflict of interest information in this publication were highlighted in a letter to the editor, but after the authors addressed the latter, the editor decided not to publish the letter as a whole. The letter was posted in its totality online by the author (68). In summary, the main problem with that paper in using it to infer the causal effect of DE on a biological response is that because the biological marker used as a marker for effect, dopamine, has an intra-individual coefficient of variation of 8.5% (69), the variability observed in the study by Milham and Stetzer (33) can be entirely explained by natural variation. Although Milham and Stetzer describe a dopamine response pattern, they describe a classical placebo effect – short-term improvement followed by return to homeostasis – as a result of non-blinding of the subjects.

An additional quantitative epidemiological study was published, but this was based on ecological correlations only (46). These analyses use 2008 national average male body mass index (BMI), fasting plasma glucose (FPG) levels, and the prevalence of diabetes obtained from other available sources and describe that when ranked, most of the countries with the highest average BMI, FPG and diabetes population prevalence are small islands; seven in the Pacific and one in the Caribbean. The non-islands in the top 10 for these metrics are Saudi Arabia, Kuwait, Jordan, and the United States. Milham stipulates that this can be explained by the fact the islands are electrified by diesel generators, which according to the author, generate a significant amount of DE, while the presence of the middle-eastern countries in the top 10 can be explained by the fact that a sizable part of their populations are off-grid and also use generators. The presence of the US in this list is explained by the fact that the US uses the earth as the major conduit for neutral return currents “allowing DE to enter homes through conductive water and sewer pipes and through the grid” (46). While the US does use grounded systems, they are operated in a way to reduce 50–60 Hz ground currents as much as possible. It is possible that high-frequency currents are less balanced and hence more prevalent in the ground system, but no evidence has been provided for this. Studies of ecological correlations can provide useful information, but they are susceptible to the “ecological fallacy”; bias that may occur because of information loss as a result of the use of aggregated data (70); while they are also susceptible to problems with correct inference as a result of residual confounding. In this case it is straightforward to alternatively explain why these small islands have comparatively high average BMI, FPG, and diabetes prevalence compared to other countries based on the distribution of standard lifestyle factors known to be associated with these biological markers, including most notably nutritional patterns and exercise (56). Similar changes toward a westernized diet associated with BMI, FPG, and diabetes have been shown to have occurred in Saudi Arabia, Kuwait, Jordan, and indeed the Middle East as a whole (71), while food patterns and trends in (increasing) portion sizes in the US are well documented (72). Although the above does not exclude DE as an explanation, it seems much more likely that distributions and changes in known lifestyle risk factors explain the observed differences. Moreover, this does not require the invention of a *hitherto* unknown, new exposure.

The same author also linked, using similar methodology with similar methodological problems (73), cardiovascular disease, cancer, suicides, and diabetes (2), as well as male life expectancy and death rates (74), to electrification rates, but these will not be discussed here because DE was only mentioned indirectly with the main exposure of interest of these studies being area electrification rates.

#### Additional Analysis: Milham and Morgan

To date, the only study that is available to quantitatively evaluate the study of the association between DE and health risk is the 2008 study on cancer risk in the teacher population of the *La Quinta* Middle School (45). In the 2010 review in Ref. (35), problems with the external comparison with the general population and issues with case definitions and exposure assessment prohibiting assessment of DE as the causal agent were highlighted. Nonetheless this study is discussed in more detail here since, although these issues remain problematic, at the same time it remains the only quantitative study that allows some assessment of the health risk of DE exposure; albeit only to a limited extent. This, relatively small, cross-sectional study is based on 18 cancers (of various sites) in 16 individuals (among 137 teachers in a middle school) and links increased risk of total cancers to employment duration and to high exposure to DE. It is unclear whether these 137 teachers are all teachers ever employed at the school, in which case a retrospective cohort study could have been designed or (more likely) was limited to those that could be contacted in which case the calculation of incidence rates is incorrect resulting in inflated risk estimates [see Ref. (75) for a discussion of an analogous study]. Exposure assessment was based on duration of employment and on DE measurements conducted on 1 day, which were linked to a roster indicating which teachers were assigned to which classrooms. DE measurements were conducted after hours and the implicit assumptions were made that exposure in the evening was similar to that during office hours as well as to other days throughout the complete time period of interest. These data were used to calculate two exposure metrics: (1) ever vs. never exposed to high exposure (arbitrarily defined as >2,000 GS units and not 50 GS units as would be expected) and (2) cumulative exposure (again arbitrarily stratified by 5,000 GS unit years classes). An important issue of cumulative exposure is the problem of interpretability because individuals with high, but short, exposure are combined with long, but low exposed individuals (and everything in between) into one metric assuming one biological mechanism (76). There are further problems with this study in that there may be some issues with unethical data acquisition, accuracy of case definitions and with whether the matching of the teachers’ population to a comparable section of the general population was done correctly (54). Milham and Morgan in response believe that their study does not require ethical approval by an appropriate review board and argue that their case definitions as well as their risk calculations are correct (77). With respect to their risk calculations, Morgan seems correct in his assertion given that a twofold increased cancer risk compared to the general population in the DE-unexposed teachers seems highly unlikely and would not point to DE as the causal factor (54). A higher incidence of specific, but not total, cancers has been observed in California state-wide (78), but subsequent analyses indicated this pattern could be ascribed to the unique distribution of known risk factors (e.g., smoking, low parity, low frequency of lactation, postmenopausal obesity, and hormone therapy use) in this population compared to the general population (79, 80). This hypothesis is further supported by the fact that an important risk factor for cervix cancer, for which California teachers have a lower than expected rate, is early intercourse and multiple sex partners; in sharp contrast to risk profiles for breast cancer (79). As such, the *La Quinta* Middle School cancer profile does not seem unique when compared to that of California as a whole, and does not point to DE as the causal factor.

To circumvent the issues of comparison with an external population, here we conduct additional internal comparisons based on data presented in the original publication (Table 2). The results indicate that statistically significant increased cancer risk is only present in the high exposed and long employed group compared to the other groups (comparisons A-C). A high increased risk is found for cumulative exposure (comparison A), but when this is split in the effect of employment length (comparison B) and the effect of DE exposure intensity (comparison C) separately, it is seen that the effect is almost exclusively the result of employment duration. This is further confirmed by the analyses of all staff exposure above 2,000 GS units and all staff employed for at least 10 years compared to low exposure (comparison G) and short employment duration (comparison H), respectively, indicating that effects observed for DE exposure intensity are most likely the result of correlation with employment length; it is straightforward to deduce that the probability of being “ever exposed to >2,000 GS units” is correlated to longer duration of employment at the school. It is further well established that age – also correlated to employment duration – is the most important risk factor for developing cancer (81), and therefore, these analyses should have been adjusted for age as an important confounder (and for other important risk factors including most importantly tobacco smoking) since these are considered standard in cancer epidemiology. This conclusion was made independently by the California Department of Health Services in an analysis of this situation (82). While they do not totally dismiss the DE hypothesis, they are similarly critical of the lack of adjustment for age or follow-up time and for presenting associations relative to the general population. As such, this re-analysis of the La Quinta middle school study provides no evidence that exposure to DE is an important causal factor in the development of cancer in this population, and our interpretation of the epidemiological evidence, as a result of these problems with the analyses of the data, are in agreement with the interpretation of the State of California Department of Health Services (82).

**Table 2:** **Re-analysis Table IV in Milham and Morgan (45)**.

Exposure group			Comparison	Comparison group		Cause^b^	OR (95%CI)
High exposed >2,000 GS	Employed +10 years	Percentage cases in group	Number	High exposed >2,000 GS	Employed +10 years		
Yes	Yes	60%^a^	A	No	No	DxI (cumulative exp)	18.0 (4.0: 81.9)
			B	Yes	No	D	21.0 (3.1–142.2)
			C	No	Yes	I	12.8 (1.8–88.4)
Yes	No	7%^a^	D	No	No	I	0.9 (0.2: 4.5)
			E	No	Yes	I vs. D	0.61 (0.08: 4.72)
No	Yes	11%	F	No	No	D	1.4 (0.3–7.6)
No	No	8%		-			
All high (>2,000 GS units) exposed		29%	Unexposed individuals			I	2.78 (0.96: 8.03)
All employed 10+ years		21%	All employed <19 years			D	4.76 (1.61–4.12)

*Raw results*.

*^a^Original paper counts individual with two primary cancers as two independent cases*.

*^b^Exposure intensity (I) or duration (D)*.

Various non-peer-reviewed sources refer to the existence of additional cases in the population who worked or were students at this middle school (60, 83). Regardless, for the benefit of furthering the argument on whether DE may have played a role in cancer development in this population, a retrospective cohort study [instead of the problematic cross-sectional cohort design (84)], including all staff employed in relevant decades, should be conducted; including proper adjustment for confounding factors including, most importantly, age.

## Discussion

In this systematic review the scientific basis for DE, or HFVTs, as an exposure metric and the available evidence on its relevance in relation to population health was evaluated.

As described, it is extremely difficult, if not impossible, to define DE as a relevant exposure metric for exposure and health studies since its measurement has evolved and comparisons between different metrics have not been established. Additionally, as DE is measured on the wiring of buildings it remains unclear how this translates to human exposure and to dose. In addition, there is no evidence in the literature that the basis for the 50 GS unit as a safety threshold is anything more than arbitrary. In fact, since the frequency band of interest lies within the RF spectrum it is unclear why DE should be a different metric at all.

With respect to health effects, it can be summarized that aside from the scientific evidence describing associations between RF radiation and health outcomes, the available evidence on human health effects of DE is based primarily on weak and flawed epidemiological evidence. More specifically, inference is mainly based on case studies, which only have a role to play in hypothesis generation and are extremely susceptible to placebo and nocebo effects, supplemented with ecological studies describing unlikely causal inferences. Evidence from studies in schools on effects on wellbeing was based on non-blinded, often uncontrolled, interventions, again extremely susceptible to placebo and nocebo effects, while evidence on cancer risk was solely based on study for which we highlighted important limitations precluding inferences being made about DE as the causal agent.

## Conclusion

Therefore, it can be concluded that despite the fact that the concept of DE is described in a number of books and references easily found on the internet, the inferences made regarding its association with increased risks on health and wellbeing are not based on scientific data of acceptable standard for such claims. This does of course not automatically imply that the concept itself is meaningless, but additional confirmatory studies will need to be conducted (and funded) to investigate whether DE could be a causal agent. To that end, several directions for future research are suggested without which, we argue, further discussion on whether DE has any effects on human health (and, what exactly DE is for that matter) is meaningless:

(1) Most importantly, DE should be properly defined in a quantitative, scientifically precise and valid manner (other than that it can be measured by a Stetzerizer^®^ Microsurge meter). Without an understanding of the exposure metric, it will not be possible to compare exposure and epidemiological studies and infer anything meaningful.
(2) The relation between DE measurements (which are measured at the mains) and actual human exposure should be established.
(3) A hypothesis for a biological mechanism of how DE could have an effect on human health and wellbeing should at the very least be proposed, but ideally should be investigated using cell or animal models to at least establish there is some merit to the claim of health risk.
(4) Further epidemiological studies using appropriate study designs (ideally double-blind, randomized, controlled trials, but for observational epidemiological studies at least these should be properly controlled for important confounding factors) should be conducted, but these will remain to be meaningless until the above points have received further attention.
(5) Despite its flaws, the most important DE study remains to be the epidemiological investigation of cancer risk in the La Quinta middle school population. Further investigation should be conducted by establishing a cohort study of the complete population of teachers (and students if this is of interest) employed during the relevant period. Of course, this will also require a better definition of the exposure and the inclusion of important confounding variables.
(6) And finally, in light of the above, publication of further case studies, especially using the methodology as published to date, is not a research direction that will result in any new knowledge.

## Author Contributions

This is a collaborative research in which both authors contributed equally to all part of the review; from conception up to submission.

## Conflict of Interest Statement

The authors declare that the research was conducted in the absence of any commercial or financial relationships that could be construed as a potential conflict of interest.

## Footnotes

^1^http://www.stetzerelectric.com

^2^http://www.webcitation.org/6eeJjDGtc

^3^http://www.emfsolutions.info/emf-faq/faqproduct-researach/25-stetzer-faq
