# Supplementary material for: Systematic Review of the Exposure Assessment and Epidemiology of High-Frequency Voltage Transients
Source: Front Public Health. 2016 Mar 29;4:52. doi: 10.3389/fpubh.2016.00052 (PMC4810027; doi:10.3389/fpubh.2016.00052)
Supplement: Supplementary file 1 [file Table_1.pdf]

Supplementary Material

Systematic Review of the Exposure Assessment and Epidemiology of High-Frequency Voltage Transients

Frank de Vocht *, Robert G. Olsen

*** Correspondence**: Dr. Frank de Vocht. School of Social and Community Medicine University of Bristol, Canynge Hall, 39 Whatley Road, Bristol, UK. BS8 2PS. Email: frank.devocht@bristol.ac.uk

List of books on high frequency voltage transients (“dirty electricity”)

Author	Title	Year of publication	edition	Publisher	Place Published	ISBN
Fisher D.	Silent Fields. *Silent Fields: The Growing Cancer Cluster Story: When Electricity Kills*	2008	1st	Lindlahr Book Publishing	Karragarra Island, Australia	978 0 6464874 3 4
Fisher D.	More Silent Fields. Cancer and the Dirty Electricity Plague. The Missing Link…	2009	1st	Joshua Books	Buddina, Australia	978 0 9806061 1 9
Fisher D.	Dirty Electricity and electromagnetic Radiation	2011	2nd	Joshua Books	Buddina, Australia	978 0 9808744 9 5
Milham S.	Dirty Electricity. Electrification and the diseases of civilation.	2012	2nd	iUniverse	Bloomington, IN. US	978 1 9389081 8 7
Fisher D.	LIGHT that HEALS Energy Medicine Today & Beyond	2014	1st	Carter Film & Book Publishing	Australia	978 0 9924129 0 6
